# Med-Aligner empowers LLM medical applications for complex medical scenarios

**DOI:** 10.1016/j.xinn.2025.101002

**Published:** 2025-06-19

**Authors:** Xiangbin Meng, Jia-ming Ji, Xiangyu Yan, Jun-tao Dai, Bo-yuan Chen, Guan Wang, Hua Xu, Jing-jia Wang, Xu-liang Wang, Da Liu, Ming-qi Zheng, Rongzhou Wu, Chuanjie Wu, Yuwei Wu, Wen-yao Wang, Zhen Song, Yaodong Yang

**Affiliations:** 1 Peng Cheng Laboratory, Shenzhen 518055, China; 2 Institute for Artificial Intelligence, Peking University, Beijing 100871, China; 3 School of Disaster and Emergency Medicine, Tianjin University, Tianjin 300072, China; 4 Tsinghua-Berkeley Shenzhen Institute, Shenzhen 518058, China; 5 Division of Emerging Interdisciplinary Areas, The Hong Kong University of Science and Technology, Hong Kong 999077, China; 6 Department of Cardiology and Institute of Vascular Medicine, Peking University Third Hospital, Beijing 100191, China; 7 Department of Cardiology, The First Hospital of Hebei Medical University, Shijiazhuang 050030, China; 8 Children’s Heart Center, The Second Affiliated Hospital and Yuying Children’s Hospital of Wenzhou Medical University, Wenzhou Medical University, Wenzhou, Zhejiang 325027, China; 9 Department of Neurology, Xuanwu Hospital Capital Medical University, Beijing 100053, China; 10 The Second Dental Center, Peking University School and Hospital of Stomatology, Beijing 100101, China

Dear Editor,

Large language models (LLMs) show great promise in medical applications, but challenges like limited high-quality data, closed-source model rigidity, and reasoning degradation during fine-tuning hinder their reliability. To address this, we present Med-Aligner, a plug-in module that learns correction residuals to improve accuracy without full model re-optimization. Trained on 267,524 anonymized medical records from 21 departments, Med-Aligner was integrated with eight LLMs (e.g., GPT-4 and Med-Llama3-8B) and evaluated on helpfulness, harmlessness, and honesty (3H). It achieved average gains of 41.3% ± 25.4%, 30.3% ± 12.4%, and 27.3% ± 14.8% in helpfulness and 10.9% ± 8.6% and 16.6% ± 11.3% in harmlessness and a median 1.7% (range: 0.4%–3.4%) improvement in honesty (*p* < 0.05). Distribution shift plots confirmed consistent gains, especially in safety and utility. Its lightweight, model-agnostic design enables deployment on resource-limited devices like smartphones. Top rankings on the Alpaca-Eval leaderboard further validate its effectiveness. By bridging open-source and proprietary LLMs, Med-Aligner offers a flexible, efficient solution for medical AI. Limitations include reliance on offline data and the need for clinical validation.

## Introduction

LLMs have shown considerable promise in advancing applications within the medical domain.^1^^,^^2^ However, the inherent complexity of medicine requires high levels of accuracy and reliability, which pose significant challenges to the effective application of LLMs.^3^ First, limited high-quality medical data during pre-training constrain LLM performance in healthcare tasks. Second, closed-source models (e.g., ChatGPT) offer minimal flexibility for parameter tuning, while open-source models (e.g., Llama series), though more adaptable, still face issues like low-quality data, high computational costs, and complex tuning processes. Parameter adjustments may also impair reasoning ability. As LLMs evolve, their outputs can contain subtle errors that are difficult even for experts to detect. RLHF (reinforcement learning with human feedback) -based methods have also reached performance bottlenecks.^4^ These challenges highlight the need for specialized modules that can augment general-purpose LLMs efficiently.

**Figure 1:**
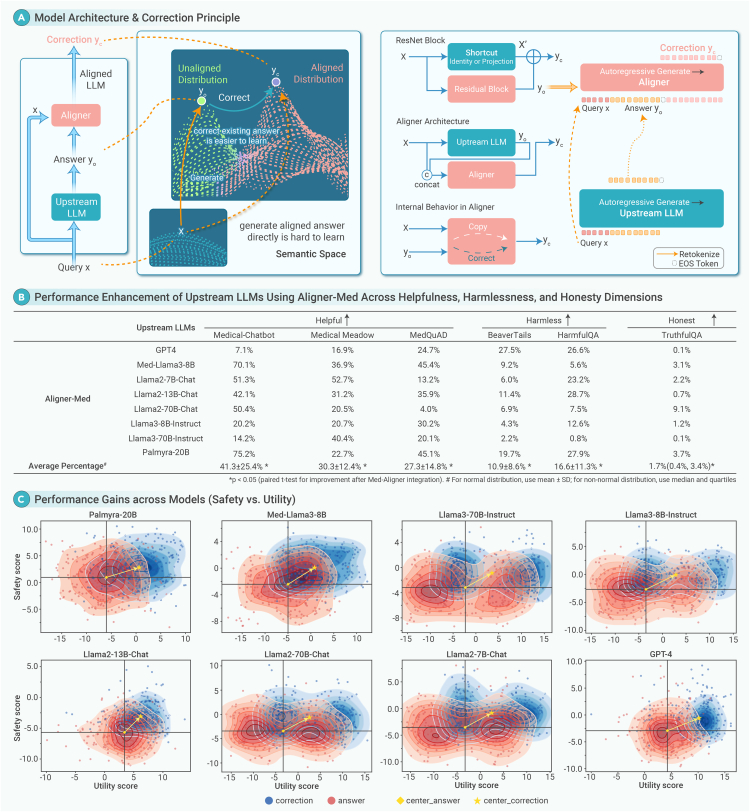
Med-Aligner enhances the performance of LLMs in complex medical scenarios. (A) Med-Aligner functions as a modular residual correction layer that aligns outputs from upstream LLMs with clinically preferred responses. It leverages supervision from expert-annotated medical records to learn correction residuals, eliminating the need for end-to-end retraining. (B) Integrating Med-Aligner into eight LLMs yields statistically significant gains in helpfulness, harmlessness, and honesty. (C) Comparisons of corrected versus uncorrected outputs show a consistent shift toward the upper-right quadrant across all models, indicating improved alignment with clinical utility and safety standards after Med-Aligner integration.

To address these limitations, we developed Med-Aligner, a plug-in alignment framework for LLMs that enables targeted medical calibration. It improves accuracy and reliability in domain-specific tasks by combining the strengths of open- and closed-source models without requiring full re-optimization (Figure 1A).

Med-Aligner learns correction residuals between preferred and non-preferred responses through algorithmic adjustments. It is a 2-billion-parameter model built using the DeepSpeed framework and transformer architecture and trained on 267,524 anonymized medical records from 21 departments (e.g., cardiology, neurology, and pediatrics). The dataset spans 4,353 disease types, with patient ages ranging from 0 to 95 and a balanced gender distribution (male: 48.7%, female: 51.3%). Annotations of preferred/non-preferred responses followed clinical guidelines and achieved 90% cross-validation agreement by licensed physicians and data scientists. The model, code, and resources are publicly available on HuggingFace (https://huggingface.co/clinic-research/Med-Aligner). We evaluated the performance gains of existing LLMs when enhanced with Med-Aligner.

## Materials and methods

This study examined three key dimensions of performance improvement in medicine: helpfulness, harmlessness, and honesty (3H). Helpfulness was assessed by the model’s ability to deliver accurate, contextually relevant, and clinically useful information for diagnosis and treatment. This evaluation was conducted using the Medical-Chatbot dataset, Medical Meadow, and MedQuAD, which together cover a wide range of clinical tasks from symptom interpretation to complex multi-system diagnostic reasoning. Harmlessness was assessed through the Beavertalis dataset and the HarmfulQA dataset, both designed to elicit and test the model’s ability to avoid generating unethical, unsafe, or misleading content. Honesty was evaluated using the TruthfulQA benchmark, focusing on the model’s ability to avoid hallucinations and preserve factual accuracy under adversarial and ambiguous conditions. Integrated scoring was conducted using reward models (for helpfulness and honesty) and cost models (for harmlessness). The final safety scores, reflecting dimensions such as harmlessness, bias, fairness, and ethics, and utility scores, reflecting helpfulness, relevance, and informativeness, were normalized to a 0–1 scale using weighted aggregation.

We evaluated the performance improvements of eight pre-existing LLMs—including GPT-4, Med-Llama3-8B, Llama2 (7B, 13B, and 70B), Llama3 (8B-Instruct and 70B-Instruct), and Palmyra-20B—after integration with Med-Aligner. Each experiment involved measuring the baseline performance of LLMs without Med-Aligner, followed by post-integration evaluations. The evaluations focused on a diverse set of clinical tasks, including disease diagnosis, treatment planning, patient management, knowledge retention, and medical reasoning. Comparative analyses were performed to quantify performance gains across the 3H dimensions. To ensure robustness, each model-dataset pair was evaluated across three independent runs using different random seeds. The final reported improvements represent the mean performance gains averaged across the three runs. Statistical significance was determined using paired t tests for normally distributed variables and Wilcoxon signed-rank tests for non-normal distributions, with *p* < 0.05 considered statistically significant.

Distribution migration plots were used to visualize performance shifts in LLMs augmented with Med-Aligner, highlighting changes in safety and utility scores between corrected and uncorrected outputs, thereby indicating overall performance improvements. These scores were derived from reward and cost models trained on human-annotated preference datasets curated by medical experts.

## Results

Med-Aligner demonstrated consistent and significant enhancements across all 3H dimensions—helpfulness, harmlessness, and honesty—when applied to eight upstream LLMs. In the helpfulness dimension, the average improvement reached 41.3% ± 25.4% (*p* < 0.05), with the largest gains observed on the Medical-Chatbot dataset from Palmyra-20B (75.2%) and Med-Llama3-8B (70.1%). On the Medical Meadow dataset, the average improvement was 30.3% ± 12.4% (*p* < 0.05), with notable gains from Llama2-7B-Chat (52.7%) and Llama3-70B-Instruct (40.4%). For the MedQuAD benchmark, the mean improvement was 27.3% ± 14.8% (*p* < 0.05), with Med-Llama3-8B (45.4%) and Palmyra-20B (45.1%) again showing strong gains. Regarding harmlessness, the average improvement on the Beavertalis dataset was 10.9% ± 8.6% (*p* < 0.05), with the highest gains observed in GPT-4 (27.5%) and Llama3-8B-Instruct (19.7%). On the HarmfulQA dataset, the mean improvement was 16.6% ± 11.3% (*p* < 0.05), with substantial gains from Llama2-13B-Chat (28.7%), Palmyra-20B (27.9%), and GPT-4 (26.6%). In the honesty dimension, the median improvement was 1.7% (range: 0.4%–3.4%) (*p* < 0.05). Llama2-70B-Chat achieved the highest individual gain (9.1%), followed by Palmyra-20B (3.7%) and Med-Llama3-8B (3.1%). GPT-4 showed a minimal change (0.1%), probably due to its already optimized baseline via RLHF (Figure 1B).

Distribution migration plots illustrated performance shifts in eight upstream LLMs after integration with Med-Aligner (2B), showing consistent improvements in safety and utility scores across all models. Med-Llama3-8B showed a strong shift toward the upper right quadrant, reflecting substantial gains. Llama2-70B-Chat exhibited a balanced but positive trend, especially in safety. Llama2-7B-Chat and Llama2-13B-Chat showed corrections concentrated in high-score regions, indicating consistent improvements. Palmyra-Med-20B displayed notable gains, particularly in safety, while Llama3-8B-Instruct showed a moderate shift favoring utility. GPT-4 demonstrated significant improvement in utility, and Llama3-70B-Instruct showed clear gains in safety, highlighting enhanced harmlessness. Overall, Med-Aligner consistently elevated model outputs across both dimensions (Figure 1C).

## Discussion

Across the core dimensions of 3H, Med-Aligner significantly enhanced the performance of both open-source and proprietary LLMs. Distribution migration plots showed consistent improvements in safety and utility scores across all tested models. For instance, Med-Llama3-8B demonstrated substantial gains in utility and safety scores, with corrected responses densely clustering in the upper right quadrant—indicative of robust alignment improvements. Similarly, Llama2-70B-Chat and Palmyra-Med-20B exhibited notable advancements, especially in safety metrics, underscoring their enhanced reliability post-alignment. These results emphasize Med-Aligner’s effectiveness across diverse model types, consistently enhancing both performance and safety.

Med-Aligner substantially improved the 3H metrics across diverse model types. While both open-source models (e.g., Med-Llama3-8B) and proprietary models (e.g., GPT-4) showed gains, the honesty improvement for GPT-4 was limited (0.1%), probably because its strong RLHF-based baseline left little room for further enhancement. Unlike RLHF or LoRA (low-rank adaptation), which require large-scale annotated datasets and significant computational resources, Med-Aligner adopts a residual correction strategy that minimizes data dependency and computational cost while preserving strong alignment performance. This made it highly efficient and well suited for medical tasks. Its plug-in architecture ensures modular flexibility, enabling model-agnostic deployment even on resource-constrained platforms such as mobile devices.

As LLMs evolve toward modular and mixture-of-experts architectures, Med-Aligner’s plug-in design supports seamless integration and lightweight adaptation.^5^^,^^6^ It is compatible with iterative upgrade frameworks such as A2A (Agent-to-Agent) and MCP (Model Context Protocol), eliminating the need for full model retraining. Med-Aligner integrates with upstream LLMs as a plug-in, eliminating the need to re-optimize medical language models from scratch while enabling functional expansion and performance enhancements in medicine. The residual correction principle enhances model accuracy, mitigates hallucination during fine-tuning, and promotes alignment with human-centric values; assists physicians with evidence-based diagnostic suggestions, reducing misdiagnoses; and generates accessible health information. Med-Aligner operates independently of expensive high-performance computing platforms and can be deployed locally on smartphones, offering exceptional flexibility and potential as a personalized AI tool for healthcare workers and enabling rapid symptom assessment for patient prioritization. Its smartphone deployment enhances accessibility in resource-limited settings (e.g., rural clinics).

## Conclusion

This study presents Med-Aligner, a lightweight plug-in framework that significantly enhances LLMs in complex medical tasks. Using residual correction, it improves 3H across diverse open- and closed-source models without full retraining. Evaluations of eight LLMs and multiple clinical benchmarks showed consistent, statistically significant improvements, especially in safety and utility. Med-Aligner’s modular, model-agnostic design enables seamless integration with upstream LLMs and deployment on resource-constrained devices like smartphones. This makes it both technically efficient and practical for clinical use. By enhancing diagnostic reasoning, reducing hallucinations, and improving response quality, Med-Aligner offers a scalable solution for medical AI.

Looking ahead, Med-Aligner is well-suited for real-time clinical decision-making, emergency triage, and telemedicine. Future work will involve prospective clinical studies to validate its real-world impact on patient care and safety. As LLMs evolve, Med-Aligner provides a robust foundation for trustworthy, specialized medical AI.

This study is limited by its reliance on offline data, lack of clinical trials, and potential annotation bias. Future work will involve real-world deployments and more diverse datasets.

## Funding and acknowledgments

This work was supported by the 10.13039/501100001809National Natural Science Foundation of China General Project (no. 623B2003), the Beijing Natural Science Foundation (no. L242135), the 10.13039/501100012166National Key Research and Development Program for Government-to-Government International Scientific and Technological Cooperation (no. 2024YFE0107100), and the “Research on Clinical Application of Medical Artificial Intelligence” Project of the Hospital Management Institute of the National Health Commission (nos. YLXX24AIA008 and YLXX24AIA026). Additional support was provided by the Hebei Province Higher Education Science and Technology Research Project (no. CXZX2025030) and the Hebei Provincial Government-funded Clinical Talent Project (no. ZF2025062).

## Declaration of interests

The authors declare no conflicts of interest.
